# Barriers to Medication Adherence and Mild Cognitive Impairment Among African Americans with Persistently Uncontrolled Hypertension: A Cross-sectional Analysis from the Southeastern Collaboration Trial

**DOI:** 10.1007/s11606-026-10477-5

**Published:** 2026-05-04

**Authors:** Sunidhi Singh, Joanna Bryan Ringel, Elizabeth Baquero, Doyle M. Cummings, Jacqueline Halladay, Andrea L. Cherrington, Lynn Andreae, Monika Safford

**Affiliations:** 1 Weill Cornell Medicine, New York, NY USA; 2 Division of General Internal Medicine, Department of Medicine, Weill Cornell Medicine, New York, NY USA; 3 Department of Family Medicine, Brody School of Medicine, East Carolina University, Greenville, NC USA; 4 Department of Family Medicine, School of Medicine, University of North Carolina, Chapel Hill, NC USA; 5 Department of Medicine, The University of Alabama at Birmingham, Birmingham, AL USA

**Keywords:** cognitive impairment, hypertension, medication adherence

## Abstract

**Background:**

Mild cognitive impairment (MCI) is frequently undetected and may lead to medication nonadherence. We examined whether more reported barriers to medication adherence could signal the presence of MCI.

**Objective:**

We developed a count of barriers (0, 1, 2, and ≥3) and determined the independent association between a greater number of barriers and odds of MCI.

**Setting:**

Rural primary care practices in Alabama and North Carolina.

**Participants:**

One thousand two hundred seventy-nine participants of the Southeastern Collaboration to Improve Blood Pressure Control trial; all were African Americans with persistently uncontrolled hypertension.

**Main Measure:**

Presence of mild cognitive impairment (MCI).

**Results:**

Participants reporting ≥ 2 barriers had 2.40 (95% CI 1.03–5.75) times higher adjusted odds of MCI compared to those reporting none; those reporting ≥ 3 barriers had 3.80 (95% CI 1.74–8.31) higher odds. As the number of barriers increased, odds of MCI increased (*p* for trend < 0.001).

**Conclusion:**

Barriers to medication adherence may signal MCI.

**Supplementary Information:**

The online version contains supplementary material available at https://doi.org/10.1007/s11606-026-10477-5.

## BACKGROUND

The prevalence of high blood pressure (HBP) among African Americans is the highest in the world, and HBP often develops much earlier and with greater severity compared to other groups.^1^ Rural counties in the Southeastern US that are populated by predominantly African American communities, sometimes characterized as the “Black Belt,” are situated within the larger “Stroke Belt” where stroke mortality is 10% higher than the rest of the country.^2^ This region is known to have some of the most under-resourced counties in the nation in terms of access to primary health care and preventive services, rates of poverty, educational attainment, and life expectancy.^3^ In rural communities throughout this geographic area, there is a much higher prevalence of hypertension-related morbidity and mortality compared to other communities.^4,5^ Practical strategies to achieve better blood pressure control in this region are needed.

Poor blood pressure control is a risk factor for, and contributor to, cognitive impairment.^6^ In addition to hypertension, residents of communities in the Black Belt region disparately suffer from additional risk factors that increase the probability of cognitive impairment including obesity, diabetes, depression, sedentary lifestyle, and low educational attainment.^7^ Mild cognitive impairment (MCI), defined by deficits in memory that do not significantly impact activities of daily living, is common in the elderly and lies on a spectrum from normal cognition to dementia.^7^ However, the diagnosis of MCI is made difficult due to the lack of systematic screening, but carries important prognostic implications due to the higher incidence of progression to dementia in patients with MCI than in patients without.^8^ A higher prevalence of cognitive impairment has also been reported in the Stroke Belt by multiple studies, and risk of dementia was found to be higher in people who were born in a high stroke mortality state, regardless of whether they relocated.^9–11^ Randomized clinical trials and prospective cohort studies have also shown that intensive blood pressure control combined with antihypertensive medications decrease the risk of developing MCI later in life.^12–14^ Moreover, there is a significant body of literature that discusses the long-term effect of persistently uncontrolled hypertension and later development of cognitive impairment.^15,16^ Effective strategies to identify early cognitive impairment in primary care would help to identify individuals for the most aggressive clinical management of hypertension to delay progression of impairment.

An underappreciated potential approach to screening for MCI could be offered by assessing barriers to medication adherence. Multiple studies have identified an association between medication nonadherence and cognitive impairment, as cognitive processes need to be intact to manage a schedule of medications.^17–21^ Lauffenburger et al. published a multicenter study quantifying adherence barriers for patients with a poorly controlled cardiometabolic condition (e.g., suboptimal hyperlipidemia, hypertension, or diabetes control along with nonadherence to a prescribed medication regimen) describing that >25% of patients experienced at least two barriers and patients with a higher number of barriers were more likely to be of minority race/ethnicity with worse disease control; the presence of each additional barrier worsened average adherence by −3.1%.^22^ Since adherence to multiple medications to control HBP is crucial, lack of optimal medication adherence could be an indicator of MCI.

We addressed this possibility using data from the Southeastern Collaboration to Improve Blood Pressure Control (SEC), a cluster-randomized trial comparing the effectiveness of two healthcare delivery innovations—practice facilitation and peer coaching—in improving blood pressure control among African American patients with persistently uncontrolled hypertension living in the rural Black Belt region.^23,24^ This particular trial population is well-suited to examine MCI because individuals with advanced cognitive impairment were excluded and MCI was methodically assessed using the Six-Item Screener (SIS), a brief and reliable method of screening participants for a large multi-arm trial for cognitive impairment and dementia in face-to-face or telephone settings.^25^ Barriers to medication adherence were also methodically assessed. We hypothesized that barriers to medication adherence that particularly require memory and cognition were associated with the presence of MCI, and that as the number of barriers increased, the odds of MCI also increased. As such, this study aimed to explore whether barriers to medication adherence could signal the need for a more comprehensive assessment of cognition and to treat risk factors for cognitive impairment more aggressively, thereby slowing the progression of cognitive decline.

## METHODS

### Study Setting: The Southeastern Collaboration Trial

Sixty-nine rural primary care practices located in Black Belt counties in Alabama and North Carolina serving predominantly African Americans with low socioeconomic status participated in the SEC Trial. The trial tested the effectiveness of peer coaching or practice facilitation, alone or in combination, on blood pressure control. The trial enrolled African American Black Belt adults (aged 19–85 years) with persistently uncontrolled hypertension (mean systolic blood pressure ≥ 140 mm Hg documented in the medical record in the preceding year and research-grade blood pressure ≥ 140/90 mm Hg at the time of screening). Details are available elsewhere.^23,24^ In brief, physician-diagnosed dementia and severe cognitive impairment were exclusion criteria. Participants completed surveys at baseline and at 6 and 12 months, and data from the baseline and 6-month survey were used here. All participating sites received approval through their respective Institutional Review Boards prior to initiation of recruitment, and all participants provided written informed consent.

### Dependent Variable: MCI

The presence of MCI was the dependent variable, assessed using the SIS, which comprised three memory and three orientation questions. Participants are told three words and asked to repeat them immediately; responses at this stage are not scored. Participants are then asked three temporal orientation questions (the day of the week, the month, and the year), with each correct response receiving one point. After 3 min of distraction, participants are asked to recall the three words, and each correctly recalled word receives one point. For a cut-point of three or more errors, the sensitivity and specificity of the SIS for cognitive impairment are 50.4% and 97.4%, respectively. We defined possible MCI at a score of ≥ 3 on the SIS at baseline or 6-month follow up.^25^ We used both the baseline and 6-month follow-up scores to make maximal use of available data.

### Main Exposure: Barriers to Medication Adherence

Barriers to medication adherence were derived from previously conducted focus groups with a similar patient population attending safety net clinics in Indiana.^26^ Participants were asked how frequently each of the 19 barriers to medication adherence occurs, with responses ranging from “never,” “rarely,” “sometimes,” “often,” to “very often” (Table 1). Participants were defined as having difficulty with an individual medication-adherence barrier if they responded with “sometimes,” “often,” or “very often.”

**Table 1:** Crude Association Between Individual Barriers to Medication and Mild Cognitive Impairment

Barrier contributing to lack of optimal medication adherence	No MCI	MCI	Crude odds ratio (95% CI)
	***N*** (%)	***N*** (%)	
N	1216	63	–
I forgot to fill my prescription in time*	375 (30.8%)	31 (49.2%)	2.17 (1.31, 3.61)
I don’t know what dose to take*	50 (4.1%)	11 (17.5%)	4.93 (2.43, 10.03)
I’m not sure exactly what each medicine is for*	140 (11.5%)	21 (33.3%)	3.84 (2.21, 6.68)
There are too many doses to take each day*	197 (16.2%)	22 (34.9%)	2.78 (1.62, 4.76)
It’s too hard to keep track of what I am supposed to take when*	121 (10.0%)	14 (22.2%)	2.59 (1.39, 4.82)
My medicines are unpleasant to take*	204 (16.8%)	21 (33.3%)	2.48 (1.44, 4.28)
I can’t afford my medicines*	287 (23.6%)	28 (44.4%)	2.59 (1.55, 4.33)
Taking medicines makes my health worse*	137 (11.3%)	15 (23.8%)	2.46 (1.34, 4.51)
I sometimes find it hard to ask my doctor or nurse questions about my medication*	109 (9.0%)	17 (27.0%)	3.75 (2.08, 6.77)
My medicines make me feel bad or have side effects I don’t like	328 (27.0%)	21 (33.3%)	1.35 (0.79, 2.32)
I have heard about side effects that I am afraid I might get	373 (30.7%)	23 (36.5%)	1.30 (0.77, 2.20)
Getting to the pharmacy to pick them up is difficult	132 (10.9%)	7 (11.1%)	1.03 (0.46, 2.30)
The pharmacy could not fill my prescription	144 (11.8%)	9 (14.3%)	1.24 (0.60, 2.57)
My doctor or nurse forgot to write a new prescription	129 (10.6%)	7 (11.1%)	1.05 (0.47, 2.36)
I ran out of medication before I could call or visit my doctor or nurse	391 (32.2%)	22 (34.9%)	1.13 (0.67, 1.93)
I don’t have enough time to talk with my doctor or nurse about problems I am having	130 (10.7%)	10 (15.9%)	1.58 (0.78, 3.17)
I sometimes forget to ask my doctor or nurse about problems that I am having	243 (20.0%)	14 (22.2%)	1.14 (0.62, 2.11)
I don’t feel my medicines are helping me	297 (24.4%)	21 (33.3%)	1.55 (0.90, 2.65)
I just don’t like taking medicine in general	509 (41.9%)	22 (34.9%)	0.75 (0.44, 1.27)

^*^Barriers to medication adherence crudely associated with MCI with *p* ≤ 0.10 were included in the medication barrier count when the barrier was reported to be present sometimes, often, or very often

### Covariates

Potential confounding covariates included age, gender, education (high school graduate versus non), income (< 20,000 vs. ≥ $20,000 annual household income^27^), social isolation (using a Patient-Reported Outcomes Measurement Information System (PROMIS) scale), total number of medications taken, physical and mental health component summary scores (PCS and MCS, respectively) of the Short Form 12-item survey assessing health status, and symptoms of depression using the eight-item Patient Health Questionnaire.^28,29^ The PROMIS Social Isolation score evaluates perceptions of being avoided, excluded, detached, disconnected from, or unknown by others, with higher scores indicating greater isolation.^30^ A score of 50 is the average for the general population of the USA; we considered any above-average score “high.” PHQ-8 scores range from 0 to 24 and were categorized into low (score of 0–4), mild (score of 5–9), and moderate-to-severe (score ≥ 10) depressive symptoms. Duration of hypertension was not available; thus, we included the baseline systolic blood pressure as a covariate. Relevant medical comorbidities included self-reported or diagnosed diabetes and cardiovascular disease; diagnoses were abstracted from medical records.

### Statistical Analysis

We examined bivariate associations between each individual barrier to medication adherence and MCI. Barriers with *p* ≤ 0.05 for the bivariate association with MCI were retained for further analysis. Using this narrower list, we developed a count of 0, 1, 2, and 3 or more barriers and described sample characteristics within each of these four categories. A logistic regression model was used to determine the association between the count of barriers to medication adherence and the presence of MCI. Both crude and fully adjusted odds ratios (OR) and 95% confidence intervals (CI) were calculated. Fully adjusted models included age, gender, education, income, social isolation, medication count, PC summary score, MC summary score PHQ, baseline systolic blood pressure, diabetes, cardiovascular disease, and site (Alabama, central North Carolina, and eastern North Carolina). In a sensitivity analysis, we modified the count of barriers to be 2, 3, or ≥ 4 compared with either 0 or 1 barrier as the reference category. The analyses above were repeated using a cut-point of ≥ 2 on the SIS to define MCI to improve sensitivity. To overcome bias due to missing data on covariates, multiple imputation with chained equations with 15 imputation datasets was used.^31^ Analyses were performed using Stata 14 (StataCorp, College Station, TX). At least one author had full access to all the data in the study and takes responsibility for its integrity and the data analysis.

## RESULTS

### Count of Barriers to Medication Adherence

Unadjusted OR for the association between each barrier to medication adherence and MCI is shown in Table 1. Nine barriers were significantly associated with MCI and were included in the medication-barrier count and are outlined in Table 1 and Supplemental Table 1. The most common barrier was not liking to take medicine in general, endorsed by 41.5% of the sample. The second most endorsed barrier was running out before being able to call the doctor (32.3%), followed by forgetting to fill the prescription on time, present in 31.7%. The greatest difference in reporting a barrier was for “I’m not sure exactly what each medicine is for,” reported by 33.3% of those with MCI but only 11.5% of those without MCI. Participants with MCI were also more likely to report being unable to afford their medicine (44.4% vs. 23.6% for those without MCI). The third largest difference was for forgetting to fill the prescription, endorsed by 49.2% of those with MCI and 30.8% of those without. With the exception of not liking taking medications in general, a higher percentage of patients with MCI endorsed each barrier compared to those without MCI. The complete results of the survey on barriers are shown in Supplemental Table 1.

### Sample Characteristics by Barrier Count

The analytic sample consisted of 1279 patients (see Supplemental Fig. 1 for exclusionary cascade). Their mean age was 57 years and 61% were female, 54% had annual income less than $20,000, and 23% had achieved less than a high school education (Table 2). On average, patients in this sample took an average of 12 medications daily with a greater number of daily medications for those with higher numbers of barriers to medication adherence. Barriers to medication adherence were reported by 61% of the cohort, with 27% reporting one barrier, 14% reporting two barriers, and 20% reporting three or more barriers.

**Table 2:** Characteristics of 1279 Participants in the Southeastern Collaboration Trial by Number of Barriers to Medication Adherence

Characteristics	All	0 barriers	1 barrier	2 barriers	≥ 3 barriers	*p* for trend*
*N*	1279	501	342	180	256	
MCI, *n* (%)	63 (4.9)	15 (3.0%)	7 (2.0%)	13 (7.2%)	28 (10.9%)	
Mean age, years (SD)	57 (12)	59 (12)	56 (12)	56 (12)	57 (12)	< 0.01
Female gender,* n* (%)	779 (60.9%)	323 (64.5%)	217 (63.5%)	91 (50.6%)	148 (57.8%)	< 0.01
< High school education, *n* (%)	286 (22.6%)	92 (18.6%)	77 (22.6%)	39 (21.7%)	78 (30.8%)	< 0.01
< $20,000 annual income, *n* (%)	612 (54.3%)	226 (49.3%)	157 (52.3%)	94 (58.4%)	135 (64.9%)	< 0.01
Social isolation**, *n* (%)	433 (34.1%)	102 (20.5%)	100 (29.2%)	79 (44.1%)	152 (60.3%)	< 0.01
Median medication count (IQR)	12 (8, 17)	11 (7, 16)	12 (8, 16)	13 (8, 18)	14 (9, 19)	< 0.01
PCS, median (IQR)	41 (31, 50)	45 (34, 52)	42 (33, 51)	39 (31, 48)	36 (28, 44)	< 0.01
MCS, median (IQR)	50 (39, 57)	53 (44, 59)	51 (40, 57)	47 (37, 56)	43 (36, 53)	< 0.01
Personal Health Questionnaire, *n* (%) Depression Scale						< 0.01
Low (score 0–4)	638 (50.0%)	317 (63.3%)	183 (53.5%)	69 (38.8%)	69 (27.0%)	
Mild (5–9)	369 (28.9%)	126 (25.1%)	101 (29.5%)	48 (27.0%)	94 (36.7%)	
Moderate to severe (≥ 10)	270 (21.1%)	58 (11.6%)	58 (17.0%)	61 (34.3%)	93 (36.3%)	
Baseline systolic blood pressure, mean (SD)	155 (17)	153 (16)	155 (15)	156 (17)	158 (20)	0.01
Diabetes (self-reported or in chart review)	596 (46.6%)	218 (43.5%)	158 (46.2%)	88 (48.9%)	132 (51.6%)	0.18
CVD (from chart review)	91 (7.1%)	28 (5.6%)	23 (6.7%)	14 (7.8%)	26 (10.2%)	0.14

*IQR*, interquartile range; *MCI*, mild cognitive impairment as indicated by a score of 3 or more on the Six Item Screener; *MCS*, Mental Component Summary Score of the Short Form 12-item health status questionnaire; *PCS*, Physical Component Summary Score of the Short Form 12-item health status questionnaire; *CVD*, cardiovascular disease; *SD*, standard deviation; *SEC*, Southeastern Collaboration to Improve Blood Pressure Control

^*^Spearman’s rho and Cochran–Armitage test were used to compute *p* for trends

^**^The PROMIS Social Isolation score assessed social isolation, see text

Approximately 5% of participants of the total sample were classified as having MCI at either baseline or 6-month follow-up; a greater proportion had MCI as the number of barriers increased. Among participants reporting at least three barriers to adherence, 11% screened positive for MCI compared to 3% in the 0 barriers group (Table 2). Compared to those with 0 barriers, a higher percentage of participants with ≥ 3 barriers had not completed high school (19% vs. 31%) and reported annual income less than $20,000 (49% vs. 65%). In addition, compared to those with 0 barriers, those with ≥ 3 barriers were more likely to be socially isolated (21% vs. 60%), have lower physical functioning (median PCS score 45 vs. 36), and mental functioning (median MCS score 53 vs. 43) and report moderate to severe depressive symptoms (12% vs. 36%).

### Number of Barriers and Odds of MCI

In the crude model, compared with participants who reported no barriers to medication adherence, the OR for MCI was 2.52 (95% CI 1.18–5.41) for participants who faced two barriers, and 3.98 (95% CI 2.08–7.60) for participants who faced at least three barriers (Table 3). This upward trend was also seen after full adjustment, where reporting at least two barriers conferred 2.43 (95% CI 1.03–5.75) times higher odds of having MCI compared to reporting no barriers. Reporting at least three barriers to medication adherence increased the odds of MCI to almost four times (OR 3.80, 95% CI 1.74–8.31). The *p*-values for trend were significant for both the crude and adjusted analyses.

**Table 3:** Association Between Number of Barriers to Medication Adherence and Mild Cognitive Impairment Among Participants in the Southeastern Collaboration Trial*

Number of Barriers	*N*	*n* with MCI (%)	Crude		Full model**	
			Odds ratio	***p***-values	Odds ratio	***p***-values
0 Barriers	501	15 (3%)	1.00		1.00	
1 Barrier	342	7 (2%)	0.68 (0.27–1.68)	0.40	0.78 (0.30–2.03)	0.62
2 Barriers	180	13 (7%)	2.52 (1.18–5.41)	0.02	2.43 (1.03–5.75)	0.04
≥ 3 Barriers	256	28 (11%)	3.98 (2.08–7.60)	< 0.01	3.80 (1.74–8.31)	< 0.01
p for trend				< 0.01		< 0.01

^*^Medication barrier count includes: I forgot to fill my prescription in time, I don’t know what dose to take, I’m not sure exactly what each medicine is for, There are too many doses to take each day, It’s too hard to keep track of what I am supposed to take when, My medicines are unpleasant to take, I can’t afford my medicines, Taking medicines makes my health worse, I sometimes find it hard to ask my doctor or nurse questions about my medication

^**^Full model adjusts for age, gender, education, income, social isolation, medication count, PCS, MCS, depressive symptoms, systolic blood pressure, diabetes, and cardiovascular disease

### Sensitivity Analyses

The results of the sensitivity analyses examining one, two, three, or four or more barriers compared to none (Supplemental Table 2), comparing two or three or more barriers to zero or one (Supplemental Table 3), and using a cut-point score of ≥ 2 on the SIS to classify individuals as having MCI (Supplemental Table 4) are consistent with the main findings. A correlation matrix of the barriers reveals correlation coefficients in the low to moderate correlation range (Supplemental Table 5). The complete survey responses are shown in Supplemental Table 6.

## DISCUSSION

We found that among a clinical trial population of African Americans with persistently uncontrolled hypertension living in the rural Southeastern US, as the number of self-reported barriers to medication adherence increased, the odds of clinically undetected MCI increased and were approximately 2.4 times greater in those reporting two barriers and nearly four times greater among those with three or more barriers, compared to those reporting none. This finding suggests that barriers to adherence to antihypertensive medications should be assessed systematically in the outpatient primary care setting to not only assist patients to overcome those barriers, but also to detect MCI in the very high-risk population with persistently uncontrolled hypertension.

While there is an extensive literature documenting an association between poor medication adherence among individuals with decreased cognitive function,^32^ our study elucidated exactly which barriers patients reported facing. We reported the prevalence of 19 different self-reported barriers to antihypertensive medication adherence, demonstrating that individuals with MCI reported more barriers to adherence compared to those who screened negative for MCI. We showed that of the nine barriers more likely to be reported by those with MCI, five rely on memory, such as forgetting to fill prescriptions and confusion about doses and the number of pills, lending face validity to the findings.

Numerous past studies have examined associations of patient characteristics with medication nonadherence, but fewer have evaluated barriers to medication adherence. Our study findings accord with Lauffenburger et al.’s findings that patients of minority race or ethnicity with worse disease control reported a higher number of barriers to medication adherence.^22,33^ Facing some type of barrier to medication adherence was very common in our sample, with over 60% reporting at least one barrier. Past studies have cited cost as a barrier to adherence,^34,35^ and we observed that while 25% of this sample overall endorsed this barrier, over 44% of those with MCI did so. Similarly, past studies have shown that pill burden creates barriers to adherence,^36^ and this was a problem for over one-third of those with MCI, compared with 16% of those without MCI.

One-third of our sample reported facing multiple barriers to medication adherence. Those with multiple barriers were more likely to have little education, low income, social isolation, a higher number of medications, low physical and mental functioning, and more severe depressive symptoms. The study population is at extremely high risk for poor cognitive outcomes given that all had persistently uncontrolled hypertension for at least a year prior to enrollment. MCI is a clinically elusive problem that can go undetected for years, and despite the exclusion criterion in the trial, undetected MCI was found to be present in 5% of the sample. Our findings suggest that patients could be screened for MCI in the primary care setting by assessing barriers to medication adherence. Studies in other populations may be warranted to confirm whether a higher number of barriers to medication adherence reported by patients with uncontrolled hypertension can serve as a potential marker for problems with cognition, prompting further assessment and aggressive control of blood pressure and other risk factors for cognitive decline.

### Limitations

Limitations to this study include the trial population and the rural Southeastern setting, both of which may limit the generalizability of the findings. Similar studies should be conducted in other areas of the country and with more typical primary care patients to confirm our findings. MCI was assessed using the SIS, which is a screening tool that lacks sensitivity but has high specificity to detect risk of current MCI. A more sensitive screening tool for MCI would be ideal for purposes of operationalizing our findings in primary care settings. Duration of hypertension was not available for incorporation into multivariable models, which may have added to residual confounding. We included baseline blood pressure in the multivariable models to offset this problem.

## CONCLUSION

In this high-risk population of patients with persistently uncontrolled hypertension, the number of reported barriers to medication adherence correlated with the likelihood of MCI. Systematically assessing barriers to medication adherence may assist in not only overcoming those barriers, but also in identifying patients at high risk of undetected MCI.

## Supplementary Information

Below is the link to the electronic supplementary material.

ESM 1 (DOCX 51.6 KB)

## Abbreviations

CI: Confidence interval
HBP: High blood pressure
MCI: Mild cognitive impairment
MCS: Mental health component summary score
PCS: Physical component summary score
PHQ-8: Patient health questionnaire – 8
PROMIS: Patient-reported outcomes measurement information system
OR: Odds ratio
SEC: Southeastern collaboration to improve blood pressure control
SIS: Six item screener
