# Supplementary material for: Barriers to Medication Adherence and Mild Cognitive Impairment Among African Americans with Persistently Uncontrolled Hypertension: A Cross-sectional Analysis from the Southeastern Collaboration Trial
Source: J Gen Intern Med. 2026 May 4;41(12):3434–40. doi: 10.1007/s11606-026-10477-5 (PMC13569766; doi:10.1007/s11606-026-10477-5)
Supplement: Supplementary file 1 [file 11606_2026_10477_MOESM1_ESM.docx]

**Barriers to medication adherence and mild cognitive impairment among African Americans with persistently uncontrolled hypertension**

Sunidhi Singh, BA^1^; Joanna Bryan Ringel, MPH^2^; Elizabeth Baquero, EdD^2^;

Doyle M. Cummings, PharmD^3^; Jacqueline Halladay, MD MPH^4^;

Andrea L. Cherrington, MD^5^; Lynn Andreae, MS^5^; Monika Safford, MD^2^

1 Weill Cornell Medicine, New York, NY, USA

2 Division of General Internal Medicine, Department of Medicine, Weill Cornell Medicine, New York, NY, USA

3 Department of Family Medicine, Brody School of Medicine, East Carolina University, Greenville, NC, USA

4 Department of Family Medicine, School of Medicine, University of North Carolina, Chapel Hill, NC, USA

5 Department of Medicine, The University of Alabama at Birmingham, Birmingham, AL, USA

**SUPPLEMENTAL DATA**

**Supplemental Table 1: Percent of Participants Experiencing Individual Barriers to Medication Adherence Sometimes/Often/Very Often, by Presence of Mild Cognitive Impairment (MCI)**

| **Barriers to Medication Adherence** | **All** | **No MCI** | **MCI** | **P-value** |
| --- | --- | --- | --- | --- |
| N | 1279 | 1216 | 63 |  |
| I forgot to fill my prescription on time. | 406 (31.7%) | 375 (30.8%) | 31 (49.2%) | 0.002 |
| I don’t know what dose to take. | 61 (4.8%) | 50 (4.1%) | 11 (17.5%) | <0.001 |
| I’m not sure exactly what each medicine is for. | 161 (12.6%) | 140 (11.5%) | 21 (33.3%) | <0.001 |
| There are too many doses to take each day. | 219 (17.1%) | 197 (16.2%) | 22 (34.9%) | <0.001 |
| It’s too hard to keep track of what I am supposed to take when. | 135 (10.6%) | 121 (10.0%) | 14 (22.2%) | 0.002 |
| My medicine is unpleasant to take. | 225 (17.6%) | 204 (16.8%) | 21 (33.3%) | <0.001 |
| I can’t afford my medicine. | 315 (24.6%) | 287 (23.6%) | 28 (44.4%) | <0.001 |
| Taking medicine makes my health worse. | 152 (11.9%) | 137 (11.3%) | 15 (23.8%) | 0.003 |
| I sometimes find it hard to ask my doctor or nurse questions about my medication. | 126 (9.9%) | 109 (9.0%) | 17 (27.0%) | <0.001 |
| My medicine makes me feel bad or have side effects I don’t like. | 349 (27.3%) | 328 (27.0%) | 21 (33.3%) | 0.269 |
| I have heard about side effects that I am afraid I might get. | 396 (31.0%) | 373 (30.7%) | 23 (36.5%) | 0.329 |
| Getting to the pharmacy to pick them up is difficult. | 139 (10.9%) | 132 (10.9%) | 7 (11.1%) | 0.949 |
| The pharmacy could not fill my prescription. | 153 (12.0%) | 144 (11.8%) | 9 (14.3%) | 0.560 |
| My doctor or nurse forgot to write a new prescription. | 136 (10.6%) | 129 (10.6%) | 7 (11.1%) | 0.900 |
| I ran out of medication before I could call or visit my doctor or nurse. | 413 (32.3%) | 391 (32.2%) | 22 (34.9%) | 0.647 |
| I don’t have enough time to talk with my doctor or nurse about problems I am having. | 140 (10.9%) | 130 (10.7%) | 10 (15.9%) | 0.199 |
| I sometimes forget to ask my doctor or nurse about problems I am having. | 257 (20.1%) | 243 (20.0%) | 14 (22.2%) | 0.665 |
| I don’t feel like my medicine is helping me. | 318 (24.9%) | 297 (24.4%) | 21 (33.3%) | 0.111 |
| I just don’t like taking medicine in general. | 531 (41.5%) | 509 (41.9%) | 22 (34.9%) | 0.276 |

**Supplemental Table 2: Association Between Number of Barriers to Medication Adherence and Mild Cognitive Impairment Among Participants in the SEC Study, comparing 1, 2, 3, and 4 or more barriers to none***

| **# Of Barriers** | **N (# MCI)** | **Crude** | | **Full Model**** | |
| --- | --- | --- | --- | --- | --- |
|  |  | **Odds Ratio** | **p-values** | **Odds Ratio** | **p-values** |
| 0 Barriers | 501 (15) | 1.00 |  | 1.00 |  |
| 1 Barrier | 342 (7) | 0.68 (0.27-1.68) | 0.400 | 0.80 (0.31-2.06) | 0.639 |
| 2 Barriers | 180 (13) | 2.52 (1.18-5.41) | 0.018 | 2.52 (1.07-5.97) | 0.035 |
| 3 Barriers | 109 (7) | 2.22 (0.88-5.59) | 0.089 | 2.33 (0.82-6.58) | 0.111 |
| ≥ 4 Barriers | 147 (21) | 5.40 (2.71-10.78) | <0.001 | 5.18 (2.18-12.31) | <0.001 |
| p for trend |  |  | <0.001 |  | <0.001 |

* Medication barrier count includes: I forgot to fill my prescription in time, I don't know what dose to take, I'm not sure exactly what each medicine is for, There are too many doses to take each day, It's too hard to keep track of what I am supposed to take when, My medicines are unpleasant to take, I can't afford my medicines, I don’t feel my medicines are helping, I just don’t like taking medicine in general

**Full model adjusts for age, gender, education, income, social isolation, medication count, physical component summary (PCS) score from the Short Form 12-item questionnaire, mental component summary (MCS) score from the Short Form 12-item questionnaire, depressive symptoms, systolic blood pressure, diabetes, and cardiovascular disease.

**Supplemental Table 3: Association Between Number of Barriers to Medication Adherence and Mild Cognitive Impairment Among Participants in the SEC Study, comparing 2 and 3 or more barriers to 0 or 1***

| **# Of Barriers** | **N (# MCI)** | **Crude** | | **Full Model**** | |
| --- | --- | --- | --- | --- | --- |
|  |  | **Odds Ratio** | **p-values** | **Odds Ratio** | **p-values** |
| 0 - 1 Barriers | 843 (22) | 1.00 |  | 1.00 |  |
| 2 Barriers | 180 (13) | 2.85 (1.41-5.78) | <0.001 | 2.67 (1.21-5.88) | 0.015 |
| ≥3 Barriers | 256 (28) | 4.14 (2.3-7.44) | <0.001 | 4.17 (2.07-8.41) | <0.001 |
| p for trend |  |  | <0.001 |  | <0.001 |

* Medication barrier count includes: I forgot to fill my prescription in time, I don't know what dose to take, I'm not sure exactly what each medicine is for, There are too many doses to take each day, It's too hard to keep track of what I am supposed to take when, My medicines are unpleasant to take, I can't afford my medicines, I don’t feel my medicines are helping, I just don’t like taking medicine in general.

**Full model adjusts for age, gender, education, income, social isolation, medication count, PCS, MCS, depressive symptoms, systolic blood pressure, diabetes, and cardiovascular disease.

**Supplemental Table 4: Association Between Number of Barriers to Medication Adherence and Mild Cognitive Impairment (MCI) Among Participants in the SEC Study using ≥2 score to classify MCI***

| **# Of Barriers** | **N (# MCI)** | **Crude** | | **Full Model**** | |
| --- | --- | --- | --- | --- | --- |
|  |  | **Odds Ratio** | **p-values** | **Odds Ratio** | **p-values** |
| 0 Barriers | 665 (74) | 1.00 |  | 1.00 |  |
| 1 Barrier | 314 (42) | 1.23 (0.82-1.85) | 0.310 | 1.13 (0.73-1.74) | 0.58 |
| 2 Barriers | 137 (27) | 1.96 (1.21-3.19) | 0.007 | 1.91 (1.13-3.25) | 0.02 |
| ≥3 Barriers | 163 (40) | 2.60 (1.69-4.00) | <0.001 | 2.01 (1.21-3.35) | 0.01 |
| p for trend |  |  | <0.001 |  | <0.001 |

* Medication barrier count includes: I forgot to fill my prescription in time, I don't know what dose to take, I'm not sure exactly what each medicine is for, There are too many doses to take each day, It's too hard to keep track of what I am supposed to take when, My medicines are unpleasant to take, I can't afford my medicines, I don’t feel my medicines are helping, I just don’t like taking medicine in general.

**Full model adjusts for age, gender, education, income, social isolation, medication count, PCS, MCS, depressive symptoms systolic blood pressure, diabetes and cardiovascular disease..

**Supplemental Table 5: Correlation Matrix for Barriers to Medication Adherence Included in the Medication Barrier Count**

|  | I forgot to fill my prescrip-tion in time | I don't know what dose to take | I'm not sure exactly what each medi-cine is for | There are too many doses to take each day | It's too hard to keep track of what I am sup-posed to take when | My medicines are unpleasant to take | I can't afford my medicines | Taking medicines makes my health worse | I sometimes find it hard to ask my doctor or nurse questions about my medica-tion |
| --- | --- | --- | --- | --- | --- | --- | --- | --- | --- |
| I forgot to fill my prescription in time | 1 |  |  |  |  |  |  |  |  |
| I don't know what dose to take | 0.23 | 1 |  |  |  |  |  |  |  |
| I'm not sure exactly what each medicine is for | 0.18 | 0.34 | 1 |  |  |  |  |  |  |
| There are too many doses to take each day | 0.19 | 0.27 | 0.33 | 1 |  |  |  |  |  |
| It's too hard to keep track of what I am supposed to take when | 0.26 | 0.34 | 0.33 | 0.40 | 1 |  |  |  |  |
| My medicines are unpleasant to take | 0.21 | 0.22 | 0.18 | 0.38 | 0.26 | 1 |  |  |  |
| I can't afford my medicines | 0.18 | 0.14 | 0.20 | 0.20 | 0.17 | 0.21 | 1 |  |  |
| Taking medicines makes my health worse | 0.10 | 0.12 | 0.12 | 0.20 | 0.14 | 0.24 | 0.14 | 1 |  |
| I sometimes find it hard to ask my doctor or nurse questions about my medication | 0.17 | 0.17 | 0.25 | 0.20 | 0.16 | 0.19 | 0.19 | 0.20 | 1 |

**Supplemental Table 6: Percent of Participants Experiencing Individual Barriers to Medication Adherence (in original Likert scale categorization), by Presence of Mild Cognitive Impairment (MCI)**

| Barriers to Medication Adherence | No MCI | MCI | p-value |
| --- | --- | --- | --- |
| N | 1216 | 63 |  |
| I forgot to fill my prescription in time |  |  | 0.03 |
| Never or Rarely | 841 (69.2%) | 32 (50.8%) |  |
| Sometimes | 297 (24.4%) | 25 (39.7%) |  |
| Often | 51 (4.2%) | 4 (6.3%) |  |
| Very Often | 27 (2.2%) | 2 (3.2%) |  |
| I don't know what dose to take |  |  | <0.001 |
| Never or Rarely | 1166 (95.9%) | 52 (82.5%) |  |
| Sometimes | 36 (3.0%) | 9 (14.3%) |  |
| Often | 11 (0.9%) | 1 (1.6%) |  |
| Very Often | 3 (0.2%) | 1 (1.6%) |  |
| I'm not sure exactly what each medicine is for |  |  | <0.001 |
| Never or Rarely | 1076 (88.5%) | 42 (66.7%) |  |
| Sometimes | 99 (8.1%) | 16 (25.4%) |  |
| Often | 24 (2.0%) | 1 (1.6%) |  |
| Very Often | 17 (1.4%) | 4 (6.3%) |  |
| There are too many doses to take each day |  |  | <0.001 |
| Never or Rarely | 1019 (83.8%) | 41 (65.1%) |  |
| Sometimes | 107 (8.8%) | 17 (27.0%) |  |
| Often | 36 (3.0%) | 2 (3.2%) |  |
| Very Often | 54 (4.4%) | 3 (4.8%) |  |
| It's too hard to keep track of what I am supposed to take when |  |  | 0.01 |
| Never or Rarely | 1095 (90.0%) | 49 (77.8%) |  |
| Sometimes | 85 (7.0%) | 8 (12.7%) |  |
| Often | 22 (1.8%) | 3 (4.8%) |  |
| Very Often | 14 (1.2%) | 3 (4.8%) |  |
| My medicines are unpleasant to take |  |  | 0.01 |
| Never or Rarely | 1012 (83.2%) | 42 (66.7%) |  |
| Sometimes | 132 (10.9%) | 15 (23.8%) |  |
| Often | 27 (2.2%) | 3 (4.8%) |  |
| Very Often | 45 (3.7%) | 3 (4.8%) |  |
| I can't afford my medicines |  |  | <0.001 |
| Never or Rarely | 929 (76.4%) | 35 (55.6%) |  |
| Sometimes | 201 (16.5%) | 18 (28.6%) |  |
| Often | 31 (2.5%) | 1 (1.6%) |  |
| Very Often | 55 (4.5%) | 9 (14.3%) |  |
| Taking medicine makes my health worse. |  |  | 0.01 |
| Never or Rarely | 1079 (88.7%) | 48 (76.2%) |  |
| Sometimes | 99 (8.1%) | 10 (15.9%) |  |
| Often | 23 (1.9%) | 2 (3.2%) |  |
| Very Often | 15 (1.2%) | 3 (4.8%) |  |
| I sometimes find it hard to ask my doctor or nurse questions about my medication. |  |  | <0.001 |
| Never or Rarely | 1107 (91.0%) | 46 (73.0%) |  |
| Sometimes | 92 (7.6%) | 11 (17.5%) |  |
| Often | 9 (0.7%) | 2 (3.2%) |  |
| Very Often | 8 (0.7%) | 4 (6.3%) |  |
| I have heard about side effects that I am afraid I might get. |  |  | 0.54 |
| Never or Rarely | 888 (73.0%) | 42 (66.7%) |  |
| Sometimes | 239 (19.7%) | 16 (25.4%) |  |
| Often | 35 (2.9%) | 1 (1.6%) |  |
| Very Often | 54 (4.4%) | 4 (6.3%) |  |
| Taking medicines makes my health worse |  |  | 0.21 |
| Never or Rarely | 843 (69.3%) | 40 (63.5%) |  |
| Sometimes | 259 (21.3%) | 12 (19.0%) |  |
| Often | 68 (5.6%) | 7 (11.1%) |  |
| Very Often | 46 (3.8%) | 4 (6.3%) |  |
| Getting to the pharmacy to pick them up is difficult. |  |  | 0.98 |
| Never or Rarely | 1084 (89.1%) | 56 (88.9%) |  |
| Sometimes | 101 (8.3%) | 5 (7.9%) |  |
| Often | 18 (1.5%) | 1 (1.6%) |  |
| Very Often | 13 (1.1%) | 1 (1.6%) |  |
| The pharmacy could not fill my prescription. |  |  | 0.74 |
| Never or Rarely | 1072 (88.2%) | 54 (85.7%) |  |
| Sometimes | 123 (10.1%) | 8 (12.7%) |  |
| Often | 10 (0.8%) | 0 (0.0%) |  |
| Very Often | 11 (0.9%) | 1 (1.6%) |  |
| My doctor or nurse forgot to write a new prescription. |  |  | 0.79 |
| Never or Rarely | 1087 (89.4%) | 56 (88.9%) |  |
| Sometimes | 110 (9.0%) | 5 (7.9%) |  |
| Often | 10 (0.8%) | 1 (1.6%) |  |
| Very Often | 9 (0.7%) | 1 (1.6%) |  |
| I ran out of medication before I could call or visit my doctor or nurse. |  |  | 0.94 |
| Never or Rarely | 825 (67.8%) | 41 (65.1%) |  |
| Sometimes | 330 (27.1%) | 18 (28.6%) |  |
| Often | 42 (3.5%) | 3 (4.8%) |  |
| Very Often | 19 (1.6%) | 1 (1.6%) |  |
| I don’t have enough time to talk with my doctor or nurse about problems I am having |  |  | 0.18 |
| Never or Rarely | 1086 (89.3%) | 53 (84.1%) |  |
| Sometimes | 102 (8.4%) | 7 (11.1%) |  |
| Often | 19 (1.6%) | 1 (1.6%) |  |
| Very Often | 9 (0.7%) | 2 (3.2%) |  |
| I sometimes forget to ask my doctor or nurse about problems I am having. |  |  | 0.86 |
| Never or Rarely | 973 (80.0%) | 49 (77.8%) |  |
| Sometimes | 205 (16.9%) | 11 (17.5%) |  |
| Often | 21 (1.7%) | 2 (3.2%) |  |
| Very Often | 17 (1.4%) | 1 (1.6%) |  |
| I don’t feel like my medicine is helping me. |  |  | 0.11 |
| Never or Rarely | 919 (75.6%) | 42 (66.7%) |  |
| Sometimes | 246 (20.2%) | 16 (25.4%) |  |
| Often | 25 (2.1%) | 1 (1.6%) |  |
| Very Often | 26 (2.1%) | 4 (6.3%) |  |
| I just don’t like taking medicine in general. |  |  | 0.62 |
| Never or Rarely | 707 (58.1%) | 41 (65.1%) |  |
| Sometimes | 213 (17.5%) | 11 (17.5%) |  |
| Often | 95 (7.8%) | 3 (4.8%) |  |
| Very Often | 201 (16.5%) | 8 (12.7%) |  |

**Supplemental Figure 1: Exclusionary Cascade Showing Construction of the Analytic Sample**

1,442

1,325

1,279

Missing information on Six Item Screener n=117

Missing information on medication barriers

n= 46

Enrolled = 1592

Baseline data corrupted

n=150
